# Case Report: Successful retrieval of a migrated left atrial appendage occluder through the synergistic use of a guidewire and a gooseneck snare

**DOI:** 10.3389/fcvm.2024.1364376

**Published:** 2024-06-04

**Authors:** Yue Bao, Hongwei Yi, Jun Ma, Hongwei Han

**Affiliations:** Department of Cardiology, Wuhan Asia Heart Hospital, Wuhan, China

**Keywords:** left atrial appendage occluder, atrial fibrillation, embolization, LACbes device, cardiac surgery

## Abstract

Left atrial appendage occluder (LAAO) dislodgement with embolization is a rare occurrence. If the LAAO migrates into the left atrium or ventricle, it can lead to acute heart failure or even death in a person, necessitating urgent surgical intervention. Currently, most cases of LAAO dislodgement are managed through open-heart surgery, while percutaneous retrieval of the LAAO has been reported only in a few cases with limited associated experience. This article reports a case of a patient in whom a migrated LACbes device was successfully retrieved using a catheter-based approach, demonstrating an innovative and minimally invasive treatment strategy.

## Introduction

Over the past two decades, there has been a sustained increase in the prevalence of patients with non-valvular atrial fibrillation (NVAF). Left atrial appendage closure (LAAC) has emerged as a safe and effective alternative to oral anticoagulants for stroke prevention, particularly in patients with a history of major hemorrhage or those at high risk for bleeding events (1). During the initial phases of LAAC adoption, there was an elevated risk associated with complications such as cardiac tamponade, air embolism, and thromboembolism; however, instances of embolism due to a dislodgement of the left atrial appendage occluder (LAAO) were infrequent. Trial data have reported a dislodgement rate with an embolization of 0.25% for the traditional WATCHMAN 2.5 device (2). When an LAAO migrates into the thoracic or abdominal aorta, it may be asymptomatic. However, if the LAAO migrates into the left atrium (LA) or left ventricle, it can cause obstruction and lead to acute heart failure or even death in a person. Therefore, immediate assessment and timely intervention are necessary for resolving this complication (3). The traditional approach to managing this issue typically involves surgical intervention, which is invasive and has a longer recovery time. Nevertheless, with advancements in interventional techniques, percutaneous retrieval of the LAAO has emerged as a viable alternative. Perrotta et al. (4) reported a case of a patient in whom a 24 French (F) sheath was used to access the LA. Initially, a 30 mm diameter loop snare was employed to secure the waist of the Amplatzer Cardiac Plug (ACP) device, ensuring its stability. Subsequently, another loop snare was used to grasp the screw of the ACP device for retrieval. However, capturing a floating device within the LA using the first loop snare can be challenging and time-consuming. Chan et al. (5) reported a case of a patient in whom two FlexCath sheaths were used for the stabilization and retrieval of a migrated ACP device; however, securing a floating ACP device with a FlexCath sheath can also be time-consuming. Against this background, we propose a novel approach: initially, a guidewire is passed through the mesh of the LACbes device, and a gooseneck snare is used to grasp the distal end of the guidewire, rapidly securing the LACbes device within the LA. Subsequently, foreign body forceps are employed to clamp the central portion of the LACbes device, allowing for its retraction into the sheath and rendering the entire procedure more straightforward, efficient, and minimally invasive.

## Case report

**Figure 1:**
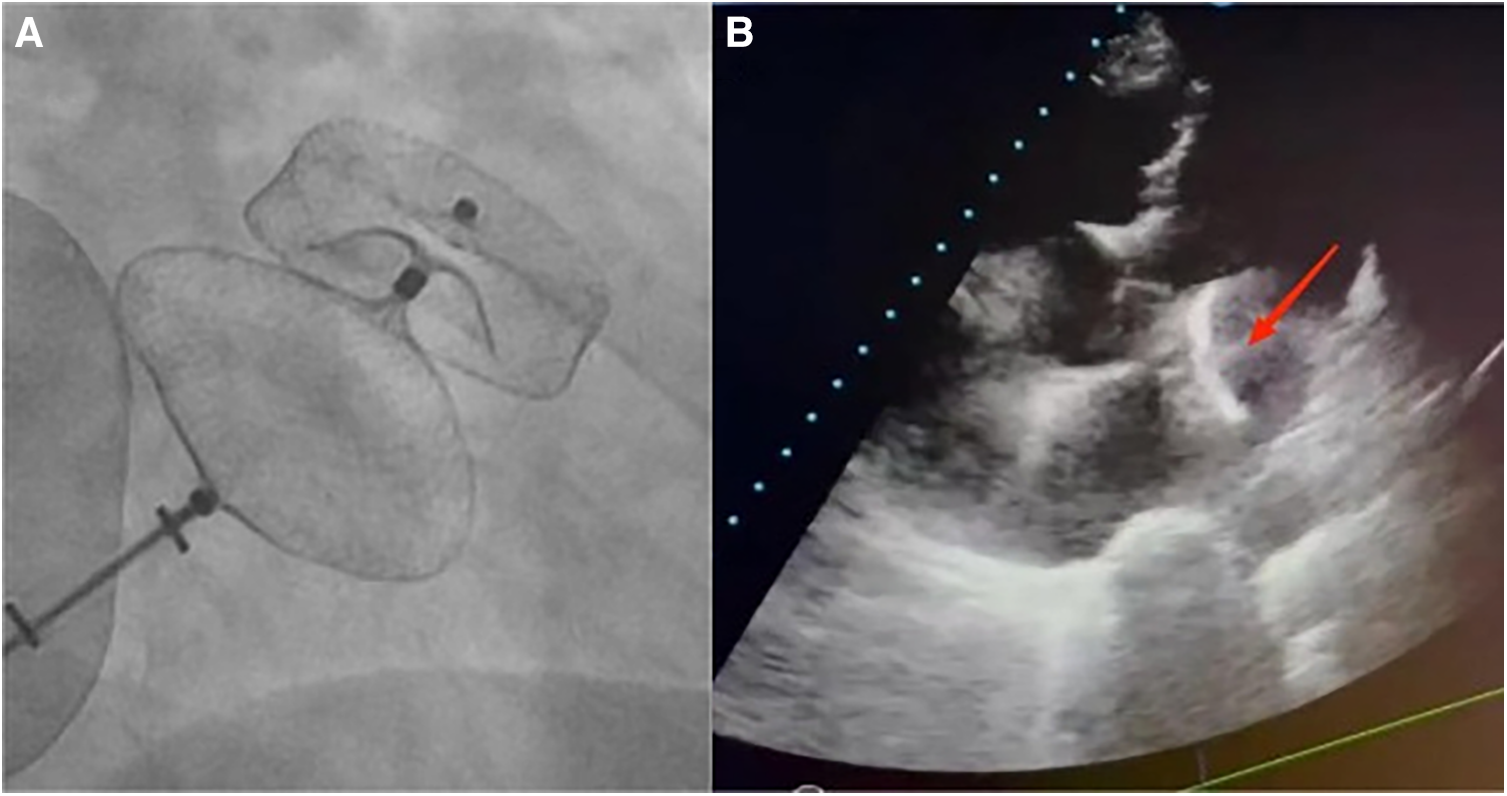
(**A**) A fluoroscopic image of a 34 mm LACbes device deployment in the LAA shows that the device is well-positioned with no significant residual shunting. Postoperative angiography confirmed this, and the tug test indicated good stability of the LACbes device. (**B**) Echocardiography reveals a migrated LAAO within the LA, with an arrow indicating its floating position.

**Figure 2:**
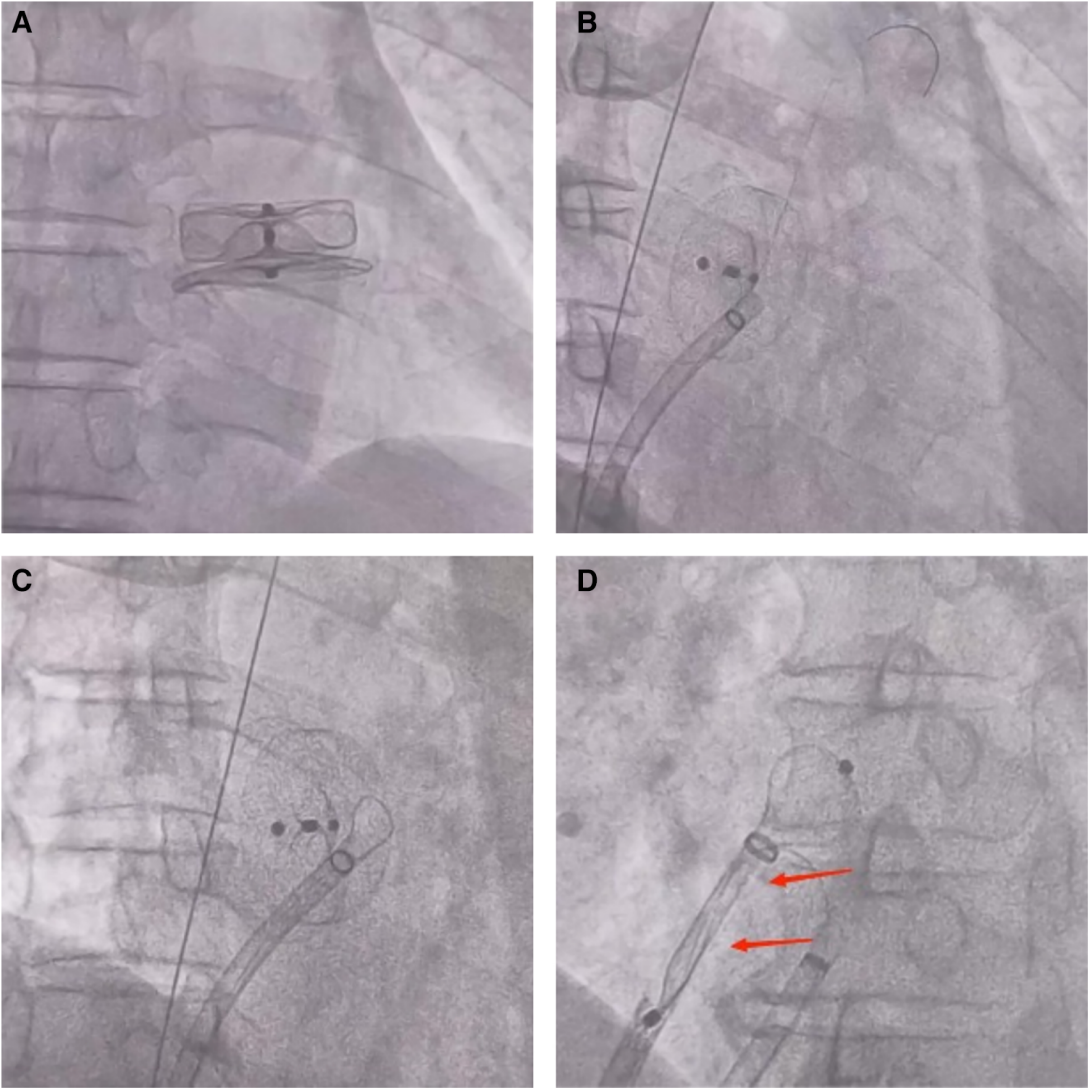
(**A**) During the performance of the procedure, fluoroscopic imaging showed that the LACbes device was observed to float in sync with the cardiac cycle within the LA. (**B**) A fluoroscopic image shows a guidewire (Runthrough NS) passing through the LACbes device mesh into the left upper pulmonary vein. (**C**) A fluoroscopic image shows the use of a gooseneck snare to capture the distal end of the guidewire (Runthrough NS), thus securing the LACbes device within the LA. (**D**) A fluoroscopic image shows the use of foreign body forceps to gently grasp and retract the LACbes device until it fully withdraws into the ProMedical 16F guiding sheath. The arrows indicate that the LACbes device has been successfully retracted into the ProMedical 16F boot sheath.

**Figure 3:**
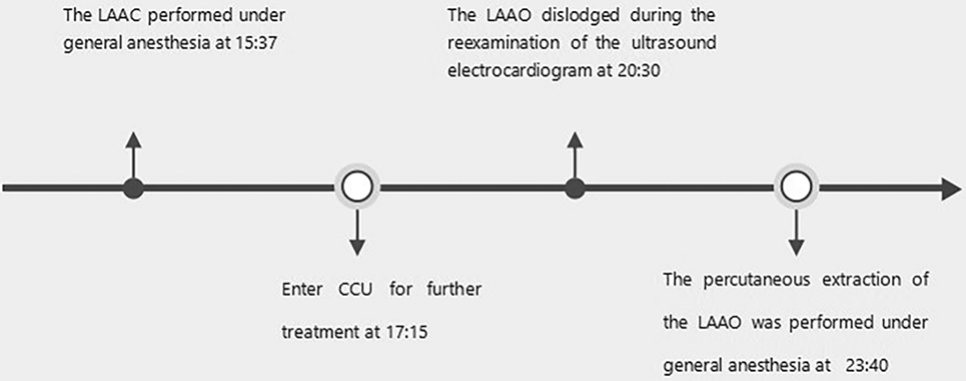
A timeline with relevant data obtained from the episode of care.

A 72-year-old man was admitted to the hospital because of palpitations that had been occurring for 5 years. Based on 7 days of continuous heart rhythm monitoring, all test results indicated atrial fibrillation. He was diagnosed with persistent atrial fibrillation. He had a history of poorly controlled hypertension for 1 year and cerebral infarction for 3 years. Upon admission, his physical examination revealed high blood pressure (170/70 mmHg), but no other abnormalities were noted. Laboratory tests also showed no significant abnormalities. The ECG showed atrial fibrillation with a heart rate of 97 beats per minute. A computed tomography scan of the head indicated a previous cerebral infarction, while echocardiography revealed left atrium dilation and moderate mitral regurgitation. Transesophageal echocardiography (TEE) showed no thrombus in the left atrial appendage (LAA). The patient presented with low-amplitude atrial fibrillation waves and significant atrial dilation, which made him unsuitable for radiofrequency ablation for atrial fibrillation. In addition, the patient was unwilling to undergo long-term oral anticoagulant therapy. The CHA2DS2-VASc score was 4 points (hypertension, age ≥65 years, and stroke history), and the HAS-BLED score was 3 points (uncontrolled hypertension, age ≥65 years, and stroke history), classifying him as being at high risk of bleeding. In accordance with the “2023 SCAI/HRS Expert Consensus on Transcatheter Left Atrial Appendage Occlusion” (6), the indication for LAAC was clear. After obtaining the patient's consent, the LAAC procedure was performed under general anesthesia on 25 September 2023. Intraoperative TEE measurements revealed a left atrial appendage occlusion zone diameter of 31.4 mm and an anchoring zone diameter of 29.4 mm. A LACbes (Shanghai ProMedical) LAAO-L 34 mm occluder was successfully implanted. Postoperative TEE and angiography revealed that the LACbes device was well-positioned with no significant residual shunting, and the tug test indicated good stability of the LACbes device (Figure 1A). The patient was then transferred to the intensive care unit. A follow-up cardiac ultrasound showed a strong echo from the occluder within the left atrium, swaying with the cardiac cycle (Figure 1B). In order to prevent the patient from developing severe hemodynamic disturbances, percutaneous LAAO retrieval was performed under general anesthesia on the same day. The LACbes device was observed floating in the LA with the cardiac cycle during the procedure (Figure 2A). The Seldinger technique was used to puncture the right femoral vein, and an Abbott Swartz L1 long sheath was inserted via the transfemoral approach. Subsequently, a transseptal puncture was performed, and, with the assistance of a guidewire, a Medtronic Flexcath steerable sheath was advanced into the LA. To clamp the center of the occluder accurately, it was necessary to stabilize the drifting LACbes device within the LA by a guidewire (Runthrough NS) through its mesh into the left upper pulmonary vein (Figure 2B). A gooseneck snare was then used to capture the distal end of the guidewire, thereby securing the LACbes device within the LA (Figure 2C). Another transseptal puncture was conducted via the femoral vein, and, with guidewire assistance, the Swartz L1 long sheath was exchanged for a 16F ProMedical guiding sheath, which was advanced into the LA. Finally, an endoscope was advanced through this sheath and foreign body forceps were used to gently grasp and retract the LACbes device until it was fully withdrawn into the ProMedical 16F guiding sheath (Figure 2D). The procedure lasted for 65 min, with the fluoroscopy taking approximately 25 min and with no contrast agent being administered during the procedure. The patient was discharged 1 week after the surgery. Despite being classified as high risk for both thromboembolic events and bleeding, the patient declined a reimplantation of the LAAO. Considering the patient's low body weight of 45 kg and high-risk score for bleeding, 15 mg of Rivaroxaban was prescribed once daily. The patient was followed up for 3 months. During this period, there were no reports of bleeding or thromboembolic events from the patient, and he was reported to be in good health. The timeline of the patient's management during hospitalization is summarized in Figure 3.

## Discussion

Migration of the LAAO is a rare but serious complication associated with LAAC procedures. However, in large-scale studies, it has been reported that the rate of incidence of LAAO migration varies from 0.2% to 0.7%, potentially related to factors such as an undersized LAAO, shallow implantation, unstable traction, or inadequate compression ratio (7, 8). Therefore, it is imperative that the operating physician possesses ample clinical experience and conducts a detailed evaluation of the cardiac structure prior to surgery in order to facilitate the selection of an LAAO of appropriate size and type and reduce the risk of LAAO detachment. The extraction of the LAAO is predominantly performed via open-chest surgery, which is highly invasive. Compared with minimally invasive procedures, open-chest surgery is associated with a longer recovery period and an increased risk of postoperative complications. Individual case reports (4, 5, 9) have described about the percutaneous removal of the LAAO. The process of capturing a floating ACP device with a loop snare or stabilizing it with a FlexCath sheath is both challenging and time-consuming. Rapidly securing a floating LAAO is considered the most challenging aspect of such surgeries.

In the case of our patient in this study, we meticulously measured the morphology, diameter, and depth of the LAA preoperatively. The patient's atypical morphology of the LAA, characterized by a larger ostium and shallower depth, meant that we deemed the intraluminal (plug-type) LAAO unsuitable. Therefore, we selected the largest available extraluminal (disc-type) LAAO. Intraoperatively, two-thirds of the LAAO's fixation disc was deployed within the LAA, while one-third extended externally. This configuration potentially increased the risk of dislodgement of the LAAO because of inadequate support force. To prevent the patient from developing acute heart failure or the condition even causing death, there was an urgent need to retrieve the LACbes device. In order to minimize invasiveness for the patient, we employed a novel procedural approach as follows: (1) A guidewire (Runthrough NS) was passed through the interstices of the LACbes device until it reached the position of the left upper pulmonary vein. Subsequently, a gooseneck snare was used to capture the distal end of the guidewire, securing the LACbes device within the LA and facilitating a precise grasping of its central part using foreign body forceps. (2) The central part of the LACbes device was clamped using the forceps and gently retracted into the guiding sheath. Passing a guidewire through the interstices of the LAAO to facilitate a more stable grasping of the LAAO, in this study, represents the introduction of an innovative surgical technique. This surgical method may be safer than others; however, in rare cases, perforation or tearing of the left atrium and migration of the LAAO into the mitral ring may occur, which would require surgical thoracotomy. This surgical approach is characterized by its efficiency, improved safety, and minimal invasiveness. However, since this method has been applied only in a single patient at our institution, further accumulation of data on this surgical procedure is necessary in order to refine and optimize it for future use.

## Data Availability

The original contributions presented in the study are included in the article/**Supplementary Material**, further inquiries can be directed to the corresponding author.
